# Chip-scale packaged in-line polarization-resolved detector for optically pumped magnetometers

**DOI:** 10.1038/s41378-026-01226-z

**Published:** 2026-03-31

**Authors:** Hui Jae Cho, Yeeun Na, Sanghyun Park, Min-Hwan Lee, Hyogi Kim, Jihyeong Ju, Dooyoung Kim, Il-Suk Kang, Jong-Bum You, Gayoung Park, Gapseop Sim, Seung-In Lee, Byung Il Lee, Soo Hyun Kwon, Jong Hyun Song, Euije Jo, Tae Hyun Kim, Giung Rang, Jae-Hyuk Ahn, Hyoungho Ko, Jongwon Lee, Jong-Kwon Lee, Geol Moon, Jongcheol Park

**Affiliations:** 1 Division of Nano Convergence Technology Service, National NanoFab Center, Daejeon, 34141 Republic of Korea; 2 Department of Electronics Engineering, Chungnam National University, Daejeon, 34134 Republic of Korea; 3 Division of Nano Convergence Technology Development, National NanoFab Center, Daejeon, 34141 Republic of Korea; 4 Department of Physics, Chonnam National University, Gwangju, 61186 Republic of Korea; 5 Center for Quantum Technologies, Chonnam National University, Gwangju, 61186 Republic of Korea; 6 Department of Laser and Optical Information Engineering, Cheongju University, Cheongju-si, Chungcheongbuk-do 28503 Korea; 7 Department of Semiconductor Convergence, Chungnam National University, Daejeon, 34134 Republic of Korea; 8 Department of System Semiconductor Engineering, Cheongju University, Cheongju-si, Chungcheongbuk-do 28503 Republic of Korea

**Keywords:** Optical sensors, Electrical and electronic engineering, Micro-optics

## Abstract

Optically pumped magnetometers (OPMs) have emerged as promising quantum sensors for neuroscience, geophysical exploration, and non-destructive testing. Their performance relies on detecting Faraday rotation of a probe beam interacting with spin-polarized alkali atoms. Conventional detection schemes based on polarizing beam splitters and discrete balanced photodetectors remain bulky and limit system miniaturization. Here, we present a chip-scale packaged in-line polarization-resolved detector (CSP-iPRD) that integrates a wire grid polarizer (WGP) and a bi-cell photodiode into a compact form factor. The integrated WGP exhibits a polarization extinction ratio (PER) of 25.3 dB at 795 nm. The assembled CSP detector, with overall dimensions of 3.5 × 3.5 × 1.8 mm^3^, achieves a common-mode rejection ratio (CMMR) of 29.6 dB and an angular resolution of 8.4 × 10^-5^ degree/Hz^1/2^. When incorporated into a spin-exchange relaxation-free (SERF) OPM, it achieves a magnetic sensitivity of 33.5 fT/Hz^1/2^ at 10 Hz. These results highlight a scalable and CMOS-compatible detection platform that paves the way for next-generation miniaturized OPMs and field-deployable quantum sensing systems.

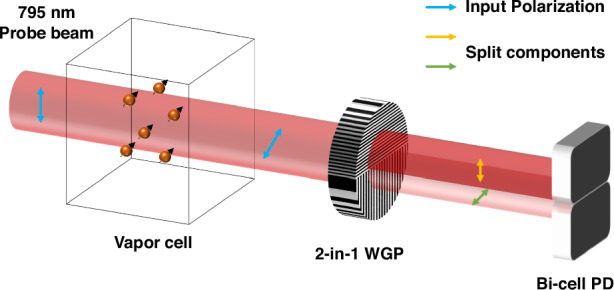

## Introduction

The rapidly growing demand for highly sensitive magnetic field measurements in the sub-femtotesla range (several fT/Hz^1/2^) has driven significant advancements in the performance of optically pumped magnetometers (OPMs)^1–3^. In particular, recent developments in micro- and nanotechnologies, including MEMS-based atomic vapor cells and semiconductor lasers, have enabled the miniaturization of OPMs^4–11^. As a result, these devices are now suitable for a wide range of applications, such as biomagnetic imaging, geophysical surveying, navigation and positioning, non-destructive testing (NDT) etc^5,12–18^. Spin-exchange relaxation-free (SERF) OPMs achieve exceptional sensitivity by detecting the polarization rotation of a probe beam interacting with alkali atoms under near-zero magnetic field conditions. They use a pumping beam tuned to atomic transitions to polarize alkali atom spins, while a probe beam that senses spin precession through polarization changes induced by external magnetic fields^1,3,7,19,20^. Traditional superconducting quantum interference devices (SQUIDs) operate at cryogenic temperatures, which introduces significant complexity, large physical size, and high operating costs^21,22^. In contrast, OPMs operate at room temperature, supporting miniaturization and enabling proximity measurements critical for high-resolution applications such as magnetoencephalography (MEG)^3,23–25^. However, conventional polarization detection methods using polarizing beam splitters (PBS) and discrete photodetectors remain bulky and limit further miniaturization and integration^26^.

Current research therefore focuses on further miniaturization and enhanced integration using micro-electromechanical systems (MEMS) technology and silicon-based meta-optics^4,6–11^. MEMS-based OPMs, in particular, have achieved sensitivities below 15 fT/Hz^1/2^, indicating robust potential for portable, compact, and even chip-scale device applications^11,27,28^. This integration can lead to more streamlined and portable measurement setups, opening the possibility for widespread clinical, industrial, and scientific applications. Recent innovations involve the application of silicon-on-sapphire (SOS) metasurface polarizing beam splitters (PBS)^6,7^. The metasurface PBS uses anisotropic rectangular meta-atoms with the same height and periodicity using the silicon. A slight variation in the lateral dimensions locally controls the transmission and phase of the probe-beam polarization components. The nanostructure is typically fabricated by e-beam lithography and diffracts the orthogonal polarizations into opposite directions^29–33^. The liquid crystals (LCs) which can modulate the amplitude, phase and polarization of electromagnetic waves, are also good candidates for the beam splitter of OPMs^34–36^. Recent studies have explored for the ultra-compact liquid crystal polarization gratings (LCPG) for differential detection^9^. LCPGs typically consist of several-micrometer-thick LC films and exhibit high polarization extinction ratios (PER) and diffraction efficiencies. These innovations also significantly reduce system size and thickness, achieving remarkable sensitivities in the order of 13.8 fT/Hz^1/2^. These advanced components significantly reduce optical complexity, device size, and enhance the integration potential of OPM systems and indicate promising directions for future developments. Nevertheless, despite the progress, these diffractive approaches typically require additional propagation distances for effective polarization separation, precise optical alignment, and specialized fabrication techniques. These requirements add complexity and hinder limiting practical and scalable integration.

To overcome these limitations, we report a chip-scale packaged in-line polarization-resolved detector (CSP-iPRD) integrated with a 2-in-1 wire grid polarizer (WGP) to measure Faraday rotation in OPMs. The CSP-iPRD integrates a 2-in-1 WGP and bi-cell photodiode to enable spatially resolved detection of two orthogonal polarization components and differential Faraday rotation readout in a compact form factor. Polarization rotation is obtained by simultaneously measuring the intensities of two orthogonal polarization components (0° and 90°) using a bi-cell photodetector integrated with the WGP. The WGP is fabricated by patterning aluminum nanowires on a quartz substrate using deep ultraviolet (DUV) lithography on a 200 mm semiconductor platform, achieving linewidths and spacings of several tens of nanometres. The PER is characterized to validate its suitability for Faraday rotation measurements. The WGP is precisely aligned and mounted onto an in-house developed bi-cell photodetector to realize a compact CSP-iPRD. The angular resolution of the CSP-iPRD is evaluated at 795 nm. Integration into a SERF OPM system demonstrates its capability for sensitive magnetic field measurements, highlighting the potential of the CSP-iPRD for advancing the scalability, miniaturization and integration of OPM systems.

## Results

### 2-in-1 wire grid polarizer (WGP)

Wire grid polarizers (WGPs) are widely used for polarization control in nanophotonic systems due to their ability to transmit TM-polarized light through subwavelength metallic slits while reflecting TE-polarized components. Their extinction performance is fundamentally governed by the grating geometry—specifically, the wire width, height, spacing, and aspect ratio relative to the incident wavelength^37–40^. We fabricated WGPs using methods tailored to target grid dimensions, material properties, and integration requirements. Achieving high extinction ratios with optimal transmittance and reflectance across a broad range of incident angles and wavelengths requires the wire width and spacing to be substantially smaller than the wavelength of the incident light, with a sufficiently high aspect ratio^41–43^.

**Fig. 1:**
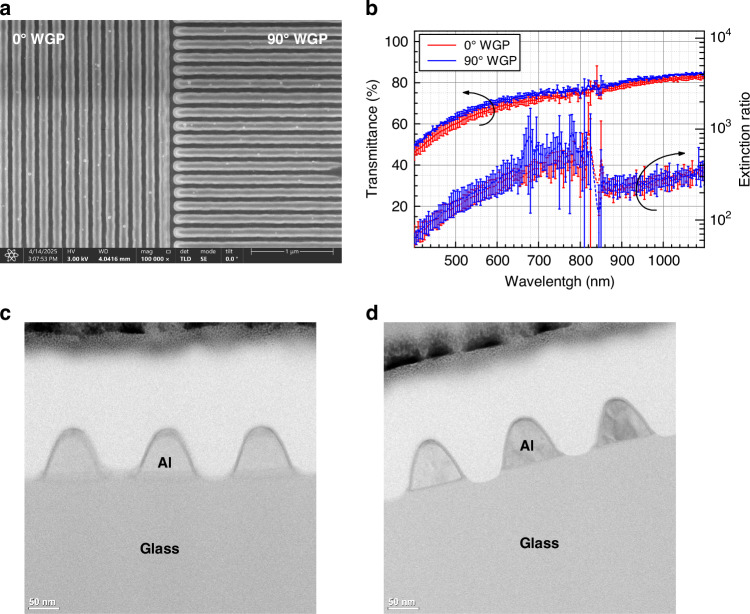
Structural and optical characterization of the fabricated 2-in-1 WGP **a** Top-view SEM image of the 2-in-1 WGP showing the 0° and 90° wire-grid regions fabricated on a single quartz substrate. **b** Measured optical performance of the WGP: co-polarized transmittance spectra for 0° (red solid line) and 90° (blue solid line), and PER for 0° (red dotted line) and 90° (blue dotted line). The arrows indicate the correspondence between the plotted curves and their respective physical quantities (transmittance and PER), which are shown on different axes, rather than highlighting specific spectral features. **c** Cross-sectional TEM image taken from the 0° wire-grid region (as defined in the top view), showing the Al nanowire grating on the quartz substrate. **d** Cross-sectional TEM image taken from the 90° wire-grid region. Here, 0° and 90° denote the polarization analysis axes defined by the WGP transmission directions, rather than the physical orientation of the metal grid lines. The scale bar represents 1 μm in (**a**) and 50 nm in (**c**) and (**d**)

The fabricated WGP structures were characterized by SEM and TEM (Fig. 1a, c, d). TEM images reveal that the aluminum wires have a convex cross-section with sidewall angles of approximately 70°, deviating from the ideal vertical profile. This sloped geometry likely limits the polarization extinction performance due to increased leakage of the TE-polarized component. The optical performance was evaluated using a spectrometer combined with a linear polarizer and a broadband source coupled to a monochromator, generating linearly polarized monochromatic light across the visible to near-infrared (NIR) range (see Fig. 1b). At 0° and 90° orientations, the co-polarized transmittances were measured as 73.8 ± 3.03% and 77.3 ± 1.67%, respectively, while the cross-polarized transmittances were 0.22 ± 0.03% and 0.16 ± 0.03%. These values correspond to polarization extinction ratios (PER) of 338.6 ± 36.8 (25.3 dB) and 507 ± 103.2 (27.0 dB), respectively.

In addition to the average PER values, the wavelength-dependent PER spectra in Fig. 1b exhibit pronounced oscillations in the 700–900 nm range. While the overall spectral trends of the measured co- and cross-polarized transmittance are consistent with electromagnetic simulations of the WGP geometry, the absolute transmittance level is consistently lower by approximately ~10% compared to the simulated results as shown in Figure S1^44,45^. This discrepancy is attributed to additional optical losses in the experimental system, including surface roughness, interface reflections, finite substrate thickness, and the absence of anti-reflection coatings, which were not included in the structural simulations.

The large amplitude of the PER oscillations can be understood from the ratio-based definition of the PER, given by PER=Tp/Ts, where Tp and Ts denote the co- and cross-polarized transmittance, respectively. In the wavelength range where the cross-polarized transmission Ts is strongly suppressed as shown in Fig. S1d, even small absolute variations in either Tp or Ts can lead to disproportionately large fluctuations in the PER. In the measurements, both Tp and Ts exhibit wavelength-dependent variations, with the effect being more pronounced for Ts in the low-transmission regime, particularly around 800–850 nm. As a result, the PER metric becomes increasingly sensitive to residual fluctuations originating from the measurement system and spectral averaging effects associated with the finite bandwidth of the monochromator-based source. Similar non-monotonic PER behavior and enhanced fluctuations in the near-infrared region have also been reported in previous studies on wire-grid polarizers, where such features were attributed to the ratio-based definition of PER and wavelength-dependent structural effects rather than to measurement artifacts^45^. These features therefore reflect the intrinsic amplification of relative variations when a ratio-based metric is evaluated under low-signal conditions.

Given that typical Faraday rotation angles in optically pumped magnetometers range from 1 to 3 milliradians, a polarizer extinction ratio exceeding 20 dB is generally required to achieve high signal-to-noise differential detection. The measured PER values of 25–27 dB are consistent with the observed convex sidewall geometry. Prior studies have shown that non-rectangular profiles, particularly those with sloped sidewalls, result in increased TE leakage and reduced extinction ratios due to incomplete field confinement^37,38,40^. Further optimization of the fabrication process—such as increasing the aspect ratio and achieving more vertical sidewalls—would be expected to enhance the PER beyond the current 25–27 dB range, thereby making the WGP more suitable for detecting sub-milliradian Faraday rotation angles in high-sensitivity OPM applications.

**Fig. 2:**
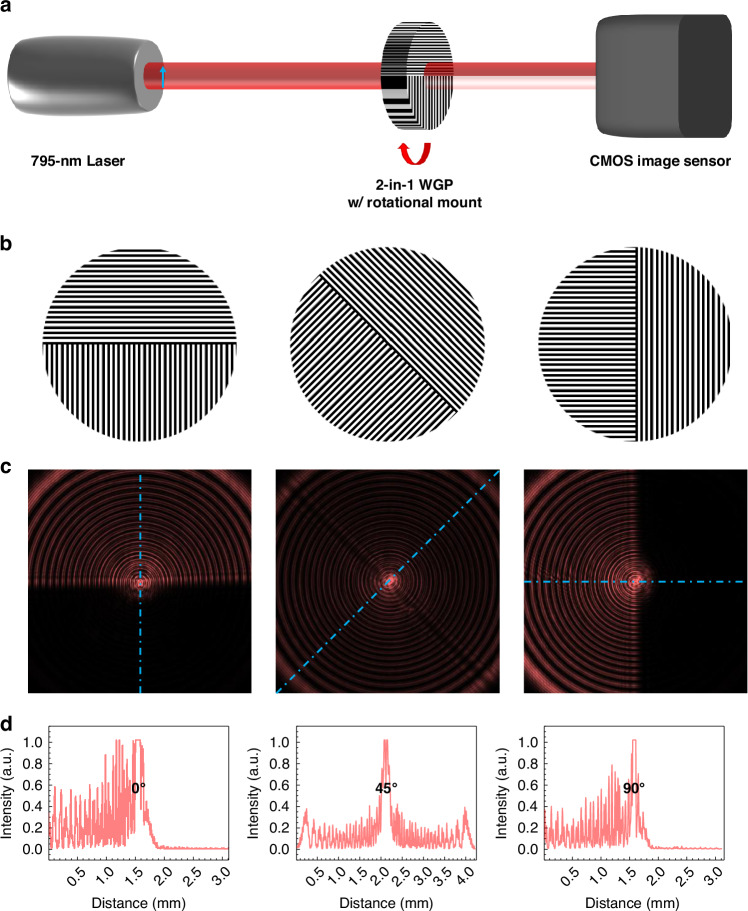
Transmission imaging of the probe beam through the 2-in-1 WGP for qualitative visualization **a** Experimental setup using a 795 nm VCSEL and a CMOS image sensor, with the WGP mounted on a rotational stage. **b** Schematic diagrams of the WGP and input polarization orientations at 0°, 45°, and 90°. **c** Transmitted images of the beam profiles for input polarization angles of 0°, 45°, and 90°, showing spatial variation in transmission intensity due to polarization-selective regions of the WGP. **d** Corresponding 1D intensity profiles extracted along the indicated direction (blue dotted line in **c**) for each polarization angle, confirming spatial separation and modulation of polarization components

To verify the spatial transmission of polarization components in the probe beam, a linearly polarized 795 nm vertical cavity surface-emitting laser (VCSEL) was directed onto the WGP, and the transmitted beam profile was imaged using a complementary metal–oxide–semiconductor (CMOS) image sensor (Fig. 2a). This measurement provides a qualitative visualization of polarization-dependent spatial transmission through the WGP, allowing direct observation of beam routing for orthogonal polarization components. As shown in Fig. 2b, Fig. 2c and Fig. 2d, the 2-in-1 WGP spatially separates the polarization components by selectively transmitting the co-polarized light, enabling measurement of polarization angle rotation. The transmitted beam profile changed significantly as the VCSEL polarization axis was rotated to 0°, 45°, and 90°, respectively. The transmitted beam profiles showed distinct bright regions corresponding to the co-polarized components, which varied with the input polarization angle. Cross-polarized regions appeared markedly dimmer, consistent with effective suppression of the orthogonal components. At 45°, the transmitted intensity became more evenly distributed between the two polarization channels, consistent with the expected superposition of components. Overall reflection exceeded 50%, primarily due to the cross-polarized regions, where reflectance above 80% was observed approximately half the area of the 2-in-1 WGP. This reflection could be further reduced by applying an anti-reflection coating in future designs.

### Bi-cell photodiode

**Fig. 3:**
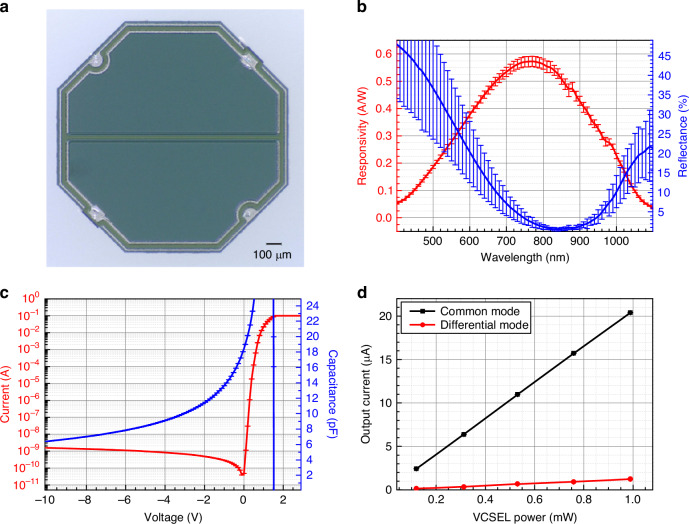
Optical and electrical characterization of the bi-cell photodiode **a** Optical microscope image of the fabricated bi-cell photodiode showing dual active areas with a 100 μm scale bar. **b** Spectral responsivity (red) and reflectance (blue) measured across the 400–1000 nm range, showing a peak responsivity of 0.56 A/W at 790 nm and a minimum reflectance of 1.4%. **c** Dark current (red, log scale) and junction capacitance (blue) as a function of reverse bias voltage. **d** Measured common-mode (black rectangle) and differential-mode (red circle) output currents as a function of incident optical power, confirming balanced detection characteristics and linear response of the bi-cell photodiode

The bi-cell photodiode was fabricated on a 200 mm silicon wafer using a standard CMOS-compatible process, and its structural and optoelectronic characteristics were evaluated. Figure3a shows a top-view optical microscope image of the fabricated device, highlighting the dual active areas of the bi-cell structure. The spectral responsivity (SR) and reflectance were measured under normal incidence using a broadband light source and monochromator, as shown in Fig.3b. The peak responsivity was 0.56 ± 0.02 A/W at 790 nm, corresponding to an external quantum efficiency of 90.96 ± 0.44%, while the reflectance at this wavelength was as low as 1.4 ± 0.7%. Although the CSP-iPRD is operated in photovoltaic mode, we characterized the dark current and junction capacitance under reverse bias to benchmark leakage and bandwidth-related characteristics. The dark current and junction capacitance were measured as a function of reverse bias voltage, as shown in Fig.3c. The dark current was 0.98 ± 0.007 nA at −5 V, and the capacitance was 8.35 ± 0.005 pF, indicating low leakage and suitable bandwidth characteristics for detection. To evaluate the electrical matching between the two photodiode units forming the bi-cell detector, the electrical characteristics were measured individually. As shown in Figure S2a, the two cells exhibit nearly identical I–V behavior over the full bias range, with closely matched dark current levels and turn-on characteristics. This high degree of matching is essential for achieving effective common-mode rejection and low-noise differential operation in the CSP-iPRD.

To assess the electrical balance of the bi-cell photodiode, common-mode and differential-mode output currents were measured as a function of total optical power from a 795 nm VCSEL (Fig.3d). The beam used in this measurement was the same as that shown in Fig. S6. The VCSEL produced a linearly polarized beam with a diameter of approximately 4.25 mm, significantly larger than the bi-cell photodiode’s active diameter of 1.5 mm, with each photodiode cell having an active area of 0.737 mm^2^. Thus, only 10.39% of the total optical power was incident on the bi-cell detector. As a result, the corresponding input optical power ranged from 12.5 to 102.8 μW. The measured differential-mode current was approximately 5.89 ± 0.36% of the common-mode signal in this experiment, and the corresponding CMMR was 24.59 dB. Since the beam exhibits a centrally peaked intensity distribution, even slight misalignment between the beam center and the bi-cell photodiode could result in unequal illumination of the two detector cells. This indicates that the differential signal likely arose from alignment-related imbalance, rather than intrinsic asymmetry of the device. These results confirm that the bi-cell photodiode maintains good electrical balance and effective common-mode rejection under realistic optical conditions.

### Chip-scale packaged in-line polarization-resolved detector (CSP-iPRD)

**Fig. 4:**
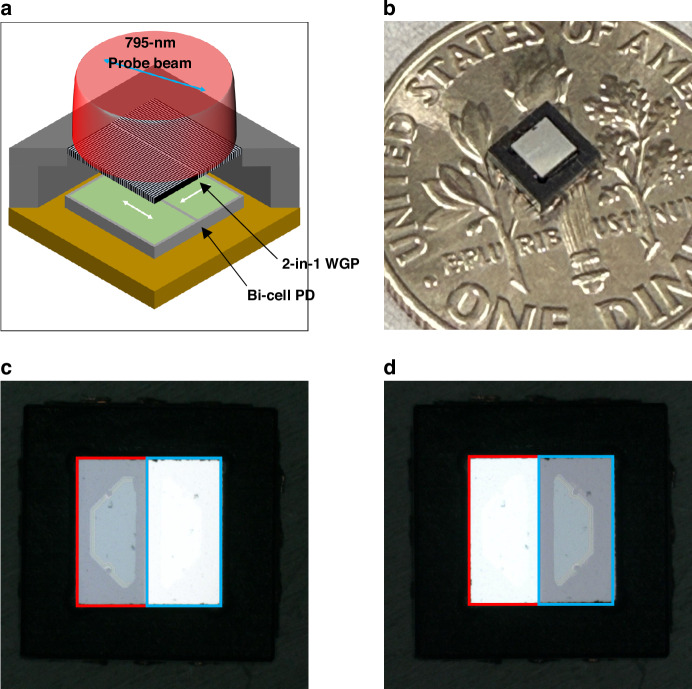
Chip-scale packaged in-line polarization-resolved detector (CSP-iPRD) assembly and spatial alignment of polarization components **a** Conceptual illustration of the CSP-iPRD integrating a 2-in-1 WGP, aluminum spacer, and bi-cell photodiode on a PCB. **b** Photograph of the fabricated CSP-iPRD with volume of 3.5 × 3.5 × 1.8 (height) mm^3^ placed on a US dime, demonstrating its compact form factor. **c,** **d** Optical microscope images of the bi-cell photodiode, showing spatial alignment corresponding to the 0° (**c**) and 90° (**d**) polarization components separated by the 2-in-1 WGP. The red and blue boxes denote the fixed physical locations of Cell A and Cell B in the bi-cell photodiode, respectively. The change in the illuminated region arises from switching the polarization state of the incident light (0° and 90°), while the WGP and photodiode remain fixed

To evaluate the optical integration of the CSP-iPRD, the assembled device was visually inspected and the spatial alignment of the polarization components to the two cells was verified. As shown in Fig.4a, the CSP-iPRD consists of a bi-cell photodiode mounted on a PCB, an anodized aluminum spacer, and a 2-in-1 WGP precisely aligned to the dual active regions. Figure4b shows the CSP-iPRD with a volume of 3.5 × 3.5 × 1.8 (height) mm^3^ placed on a U.S. dime, demonstrating its compact, chip-scale form factor. Figure 4c and Fig. 4d display microscope images of the beam spots projected onto each active area through the integrated WGP. The spatially separated polarization components—0 ° and 90 °—were aligned to illuminate the individual cells (Cell A and Cell B), confirming successful polarization-resolved beam delivery through the chip-scale packaging process. Fixed surface features (e.g., dust particles on the WGP) appear at identical positions in both images, confirming that the same photodiode cells are observed and that the change in visible illumination arises solely from polarization-dependent transmission rather than from misalignment or imaging artifacts. These observations validate the spatial integrity of the packaged detector prior to system-level characterization.

**Fig. 5:**
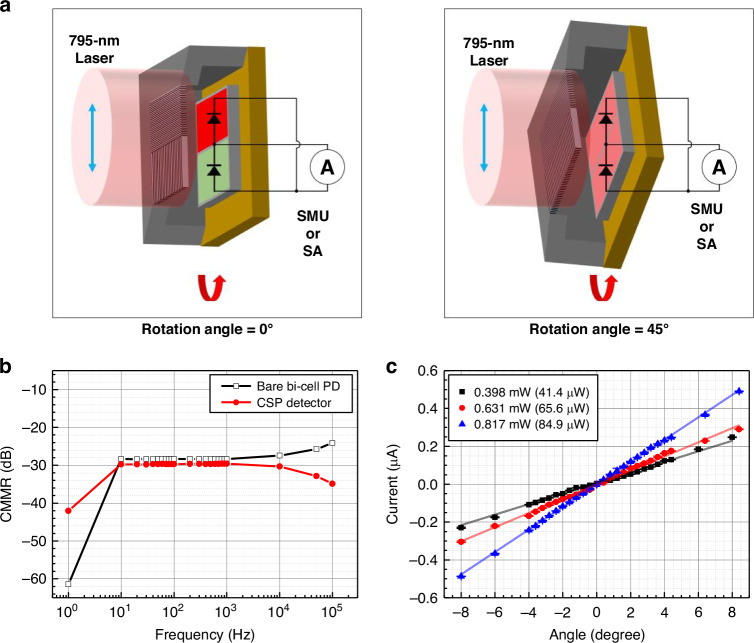
Angular response and common-mode rejection performance of the CSP-iPRD **a** Experimental setup for characterizing angular sensitivity and common-mode rejection. A linearly polarized 795 nm beam is directed through a collimator and incident on the CSP-iPRD mounted on a rotational stage. The common-mode output is measured at 0° (red cell, left), and the differential-mode output at 45° (pink cell, right). **b** Comparison of common-mode rejection ratio (CMMR) as a function of modulation frequency between the bare bi-cell photodiode (open circle) and the CSP-iPRD (filled rectangle). The results show that chip-scale integration does not significantly degrade the CMMR, maintaining high suppression across 1 Hz to 10 kHz. **c** Differential photocurrent as a function of polarization rotation angle (symbols), measured at incident optical powers ranging from 41.4 μW to 84.9 μW

The CSP-iPRD, integrating the 2-in-1 WGP and bi-cell photodiode, was characterized for system-level performance including angular resolution and common-mode rejection behavior (Fig. 5a). As illustrated in Fig. 5a, the CSP-iPRD was mounted on a fixed optical axis and irradiated with a collimated 795 nm VCSEL beam. The beam used in this measurement was the same as that shown in the Fig. S6, and exhibited a centrally peaked intensity profile. The WGP and photodiode were packaged as described previously.

The CMMR of the CSP-iPRD was evaluated by illuminating the detector with co-polarized light and measuring the differential output current over a range of modulation frequencies. Figure S2b shows the measured magnitude of common-mode and differential-mode output as a function of the modulation frequency applied to the VCSEL, confirming that the differential signal remains effectively suppressed up to 10 kHz. As shown in Fig. 5b, the CMMR of the bare bi-cell photodiode, measured without the WGP, was 28.3 dB at frequencies up to 1 kHz. The magnitude degradation observed beyond 10 kHz is attributed to the frequency limitation of the laser diode current driver, which has a cut-off frequency of 150 kHz. To evaluate the CMMR of the CSP-iPRD, the device was rotated to polarization angles of 0° and 45°. The 0° orientation yields the maximum output current, corresponding to the common-mode response, while the 45° orientation produces the minimum output, representing the differential-mode signal. The CMMR of the CSP-iPRD was measured to be 29.6 dB, comparable to the bare photodiode (Fig. 5b). The results show that the CMMR remains stable across the tested frequency range, indicating that the chip-scale packaging does not significantly degrade common-mode suppression performance. The small difference between the CSP-iPRD and the bare bi-cell photodiode is attributed to minor alignment errors during measurement, rather than an inherent limitation of the packaging.

The angular response of the CSP-iPRD was evaluated by measuring the differential photocurrent as a function of the polarization rotation angle of the input probe beam (Fig. 5c). The incident optical power was controlled by adjusting the drive current of the VCSEL and ranged from 0.398 mW to 0.817 mW. Considering the beam diameter and the detector’s active area, the corresponding optical power incident on the CSP-iPRD was estimated to be 41.4–84.9 μW. The angular resolution as a function of incident optical power is summarized in Supplementary Table S1. Linear fitting of the measured response at 84.9μW yielded a slope of 59.31 nA/degree, an intercept of –3.1 nA, and a coefficient of determination (R^2^) of 0.999. The angular responsivity normalized to incident optical power was 0.698 nA∙μW^-1^∙deg^-1^. The CSP-iPRD maintained excellent linearity over a dynamic range of ±8 °, confirming its capability to detect minute Faraday rotation signals at a probe wavelength of 795 nm. Given this angular resolution and the input-referred current noise of the transimpedance amplifier (5pA/Hz^1/2^ for the TIA400, Koheron), the CSP-iPRD can reliably resolve polarization rotation angles below 1mrad, making it suitable for detecting sub-milliradian Faraday rotation in practical OPM systems. As summarized in Supplementary Table S1, the angular resolution improves from ~1.8 × 10^-4^ to ~8.4 × 10^-5^ degree/Hz^1/2^ as the incident optical power increases from 41.4 to 84.9 μW, with an intermediate value of ~1.3 × 10^-4^ degree/Hz^1/2^ at 65.6 μW. This trend is consistent with shot-noise-limited scaling, which is used here as the theoretical benchmark to quantify the impact of reduced optical power.

### Magnetic sensitivity in SERF OPM

**Fig. 6:**
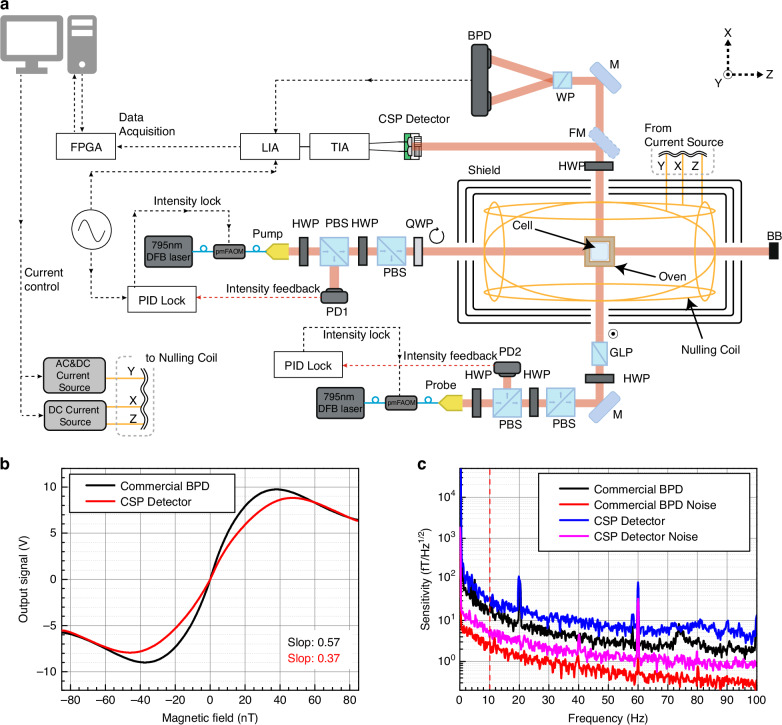
Integration of the CSP-iPRD into a spin-exchange relaxation-free (SERF) optically pumped magnetometer (OPM) and magnetic field sensitivity characterization **a** Schematic of the SERF OPM system incorporating pump and probe lasers, an alkali vapor cell with magnetic shielding, and balanced optical detection using the CSP-iPRD. The inset illustrates a conventional detection scheme using a polarizing beam splitter (PBS) and a commercial balanced photodetector (BPD). **b** Output signal versus applied magnetic field for the commercial BPD (black) and the CSP detector (red), showing polarization rotation-induced modulation with signal slopes of 0.57 and 0.37, respectively. **c** Measured magnetic noise spectra under zero-field conditions for the commercial BPD (black) and CSP-iPRD (blue), yielding a sensitivity of 33.5fT/Hz^1/2^ at 10 Hz. The intrinsic noise floors of the commercial BPD (red) and CSP-iPRD (magenta) are also shown

To validate the performance of the CSP-iPRD within a functional OPM system, the detector was integrated into a SERF OPM setup as shown in Fig. 6a. Figure 6a illustrates the experimental setup for a SERF magnetometer operating in AC-mode, consisting of a pump beam and a probe beam incident vertically onto the vapor cell, and the intensity of the pump beam is periodically modulated at 1 kHz by an acousto-optic modulator^46^.

A 795-nm linearly polarized laser and a circularly polarized laser were used as the probe and pump, respectively, and each beam was directed through a rubidium vapor cell containing the isotopically enriched ^87^Rb and N2 (700 Torr) under SERF conditions. The differential signal from the CSP-iPRD was monitored to detect the polarization rotation induced by the spin precession of the alkali atoms in response to applied magnetic fields. The differential output from the CSP-iPRD was routed via an SMA connector to a low-noise current preamplifier (SR570, Stanford Research Systems) for further signal conditioning. The resulting signal was processed using a lock-in amplifier (LIA) and recorded by a data acquisition system (DAQ) for spectral analysis. In this system, fluctuations in the intensity and polarization of the pump and probe beams are the dominant limiting factors for magnetic sensitivity. To minimize these effects, each laser beam passes through a polarizing beam splitter (PBS), converting polarization fluctuations into intensity fluctuations. A portion of the beam is then picked off using a 9:1 beam splitter (not shown in the setup) to perform the laser intensity locking. Additionally, to minimize 60 Hz electrical noise, all power supplies are connected to an uninterruptible power supply (UPS), and the UPS is isolated from the external power source during the sensitivity measurement procedure.

For reference, we first measured the SERF OPM performance using a commercial balanced photodiode (PDB210A, Thorlabs). The polarization rotation induced by the magnetic field causes an imbalance between these two components, which is detected using the balanced photodiode (PDB210A, Thorlabs). The resulting differential signal corresponds to changes in the external magnetic field along the y-axis, producing a dispersive signal as shown in Fig. 6b. When the y-axis magnetic field is zero, the dispersive signal also becomes zero. The slope of the dispersive signal at this point is obtained, and the power spectral density of the differential signal is measured using a spectrum analyzer to determine the magnetic sensitivity. The magnetic field sensitivity was evaluated by analyzing the power spectral density obtained from the resulting differential photocurrent signal and the slope of the signal near zero magnetic field. With these optimizations, the measured magnetic sensitivity reaches 16.1 fT/Hz^1/2^ at 10 Hz and the noise floor of the BPD is 2.44 fT/Hz^1/2^ at 10 Hz, as shown in Fig. 6c.

The magnetic field sensitivity and slope of the signal near zero magnetic field were also evaluated by using the proposed CSP-iPRD as shown in Fig. 6b and Fig. 6c. The CSP-iPRD-based SERF OPM achieved a magnetic sensitivity of 33.5 fT/Hz^1/2^ at 10 Hz, and the intrinsic noise floor of the CSP-iPRD and associated electronics was measured to be 5.07 fT/Hz^1/2^ at 10 Hz which is sufficiently low for high-sensitivity magnetic field measurements. The sensitivity performance is primarily limited by magnetic noise from the shielding (~10 fT/Hz^1/2^).

The reduced sensitivity of the CSP-iPRD compared to the conventional scheme is largely attributed to lower detected optical power, resulting in a degradation of SNR as predicted by the fundamental shot-noise-limited benchmark^1,47–49^. As shown in Fig.6c, the CSP-iPRD exhibits a sensitivity approximately ~2× worse than that of the conventional detection scheme, due to reduced optical throughput from the integrated WGP. In the CSP-iPRD, only the transmitted polarization components are collected (the reflected components are not detected), and the measured WGP transmittance was 73.8 ± 3.03% and 77.3 ± 1.67% for co-polarized light aligned with 0° and 90° orientations, respectively, resulting in an effective optical power of ~37.7% relative to the total probe beam. This reduction leads to an SNR decrease by a factor of √0.377 ≈ 0.614 and a corresponding ~1.6× degradation in sensitivity compared to an ideal PBS-based system.

In addition to the loss from polarization filtering, the limited aperture size of the CSP-iPRD also contributes significantly to sensitivity degradation. The probe beam follows a Gaussian profile with a 1/e^2^ diameter of 2.8 mm, while the bi-cell photodiode in the CSP-iPRD has a diameter of 1.5 mm. Based on the Gaussian intensity distribution, approximately 43.6% of the optical power falls within the detector area. Considering polarization selectivity (×0.5) and the average WGP transmittance (75.5%), the net optical power captured is ~16.5% of the total probe beam. This further reduces the SNR by a factor of √0.165 ≈ 0.406, resulting in a total sensitivity degradation of ~2.46× compared to an ideal full-aperture, dual-polarization detection scheme.

To further clarify the noise contributions, we evaluated the noise-equivalent power (NEP) of the detector system. The photodiode operates in photovoltaic (zero-bias) mode with a responsivity of 0.567 A/W at 795 nm. Assuming an effective shunt resistance on the order of 10^8^ Ω, the corresponding Johnson current noise is ~10–15 fA/Hz^1/2^, yielding an intrinsic photodiode NEP of ~20–30 fW/Hz^1/2^. In contrast, the system NEP is dominated by the front-end transimpedance amplifier (TIA), whose input current noise (~5 pA/Hz^1/2^) results in a system NEP of ~8.8 pW/Hz^1/2^. Therefore, under the reduced optical power conditions (~16.5% probe transmission), the CSP-iPRD operates in an electronics-noise-limited regime rather than a strictly shot-noise-limited one. The shot-noise-limited scaling is thus used as a theoretical benchmark to quantify the impact of reduced optical power. Notably, although the commercial balanced photoreceiver used in the conventional scheme (PDB210A) has a lower specified NEP ( ~ 2.2 pW/Hz^1/2^) than our readout chain (~8.8 pW/Hz^1/2^), the measured sensitivity degradation is smaller (~2×), suggesting that the SERF-OPM sensitivity at 10 Hz is dominated by system-level technical noise and shielding-related magnetic noise rather than detector NEP alone.

Despite this reduction, the monolithic integration of the WGP enables a compact, alignment-free, and CMOS-compatible platform, making the CSP-iPRD highly attractive for chip-scale OPM systems where size, robustness, and manufacturability are critical.

## Discussion

The integration of the 2-in-1 WGP and bi-cell photodiode into a CSP-iPRD highlights a path towards miniaturized, high-sensitivity OPMs. The bi-cell photodiode exhibited high responsivity, low dark current, and excellent CMMR. The CSP-iPRD demonstrated a volume of 3.5 × 3.5 × 1.8 (height) mm^3^ and maintained high angular sensitivity and system-level common-mode suppression despite minor alignment imperfections. The achieved magnetic sensitivity of 33.5fT/Hz^1/2^ indicates that the CSP-iPRD provides a viable alternative to conventional free-space balanced detection setups, significantly simplifying OPM system design. The combination of small form factor, low noise, and high rejection of common-mode disturbances offers a pathway for developing portable and wearable magnetometers.

A comparison of various polarization detection schemes used in OPM systems is summarized in Table 1, which consolidates the key performance trade-offs associated with miniaturization. While metasurface-based PBS structures offer submicron-scale optical components, their extinction ratios and fabrication yield are still limited by tight lithographic requirements and alignment sensitivity. Liquid crystal polarization gratings (LCPGs) achieve excellent angular resolution and extinction but require precise multilayer stacking and are not readily compatible with CMOS processes. In contrast, the WGP-based CSP-iPRD proposed in this work represents an intentional trade-off between optical throughput and system integration. Although the CSP-iPRD exhibits a reduced sensitivity compared to conventional free-space PBS-based detection schemes, the achieved magnetic sensitivity of 33.5 fT/Hz^1/2^ remains sufficient for a broad range of practical SERF-OPM applications. For example, magnetocardiography (MCG) and non-destructive testing (NDT) typically require sensitivities on the order of 50–100 fT/Hz^1/2^, while emerging compact and wearable MEG systems often operate in the 20–50 fT/Hz^1/2^ range by leveraging sensor proximity, array-level averaging, and differential noise suppression. In these scenarios, the benefits of chip-scale integration—including reduced footprint, improved mechanical robustness, and simplified optical alignment—can outweigh the penalty in single-sensor sensitivity.

**Table 1:** Comparison of optical polarization detection schemes used in optically pumped magnetometers (OPMs)

Category	Feature	Commercial PBS	Metasurface PBS [6]	LCPG-based [9]	This work (CSP-iPRD)
Physical & Integration	Device thickness	>10 mm^†^	~500 nm^†^	Several μm^†^	1.8 mm
	Alignment robustness	Low	Moderate	Low	High (Alignment-free)
	Integration level	Free-space	Hybrid	Hybrid	Chip-scale (Monolithic)
	CMOS compatibility	No	Partially	Limited	Yes (Full)
Optical Specifications	Optical geometry	Free-space split	±1st order diffractive	Same-side diffraction	Inline, co-planar
	Extinction ratio (PER)	~ 30 dB	15 ~ 20 dB	35.6 dB	25.3 dB
	Optical throughput^††^ (Ideal)	100%	100%	100%	50%
	Actual efficiency (Current setup)	-	~90%	99%	16.5%
Sensor Performance	Angular resolution	-	-	~8.5 × 10^-3^°	8.4 × 10^-5^ °^†††^
	Magnetic sensitivity	~16.1fT/Hz^1/2^ (10 Hz)	~300fT/Hz^1/2^	13.8–14.9fT/Hz^1/2^	33.5fT/Hz^1/2^ (10 Hz)

Values are based on reported experimental results. CPER: contrast PER; ^†^Device thickness excludes the detector thickness. ^††^Expected under ideal coupling and aperture matching; current setup yields ~16.5%. ^†††^Calculated from noise-equivalent angular resolution of 8.4 × 10^-5^ °/Hz^1/2^ at 84.9 µW and 1 Hz bandwidth. The noise-equivalent angular resolution was estimated from the measured angular responsivity and the input-referred current noise of the transimpedance amplifier (5 pA/Hz^1/2^). The reported range corresponds to incident optical powers of 41.4–84.9 µW (see Supplementary Table S1)

In addition, the CSP-iPRD architecture is inherently compatible with balanced and gradiometric detection schemes, which are essential for suppressing environmental magnetic noise in practical MEG systems. In such configurations, the effective system sensitivity is enhanced through common-mode rejection and differential readout rather than relying solely on the intrinsic noise floor of an individual sensor. Combined with the reduced sensor-to-source distance enabled by chip-scale packaging and the scalability toward dense sensor arrays, the demonstrated sensitivity is sufficient to support MEG measurements in compact and wearable implementations. At the same time, the monolithic and co-planar integration of the WGP and bi-cell photodiode significantly relaxes alignment requirements and enables a compact, CMOS-compatible chip-scale architecture. These advantages also enable favorable cost scalability at the system level by leveraging mature semiconductor manufacturing processes and eliminating discrete optical components and alignment-intensive assembly steps. This balance between moderate sensitivity degradation and substantial gains in robustness, scalability, and manufacturability underscores the suitability of the CSP-iPRD for next-generation chip-scale OPM systems.

Although the current experimental setup exhibits a reduced net optical throughput due to finite detector aperture and beam-size mismatch, optimized coupling and aperture matching are expected to improve the throughput to ~50% under ideal conditions, primarily limited by single-polarization detection and WGP transmittance. Future work will focus on implementing anti-reflection coatings to further enhance optical throughput, optimizing beam coupling to minimize aperture-related losses, and exploring integration with microfabricated vapor cells to realize a fully chip-scale OPM. Investigating long-term stability and environmental robustness of the CSP-iPRD will also be essential for practical deployment in clinical, geophysical, and industrial applications.

## Methods

### Fabrication of the 2-in-1 wire-grid polarizer (WGP)

The 2-in-1 WGP was fabricated using a 200 mm wafer process based on deep ultraviolet (DUV) lithography and plasma etching techniques. A 4× reticle, with feature sizes four times larger than the target dimensions, was used to define the nanowire structures, aiming for a final linewidth and spacing of 80 nm each. A quartz wafer was thoroughly cleaned, followed by sequential deposition of a 10 nm titanium adhesion layer and a 140 nm aluminum layer to form the wire material. Lithography was performed using an ArF-Dry scanner (XT1250D, ASML) operating at 193 nm wavelength, achieving precise patterning of the WGP structures. To enhance pattern resolution, a 30 nm-thick bottom anti-reflective coating (BARC) was spin-coated onto the wafers, followed by a 140 nm-thick photoresist layer. After soft baking, 80 nm lines with a 160 nm pitch were patterned using the scanner, and post-exposure bake (PEB) and hard bake steps were performed. Following photoresist development, the exposed BARC was etched using inductively coupled plasma reactive ion etching (ICP-RIE). The aluminum and titanium layers were then etched using a conductor reactive ion etcher (DPS, Applied Materials) under specific process conditions. The chamber pressure was maintained at 8 mTorr, with a source power of 800 W and a bias power of 140 W. The process gases used were Cl_2_ (80 sccm), BCl_3_ (40 sccm), and C_2_H_4_ (20 sccm) at a process temperature of 40 °C. Residual photoresist was removed using a dry PR stripper (DAS-2000, PSK) operated at 1000mTorr, 2500 W microwave power, and 250 °C for 60 s in an O_2_/N_2_ atmosphere. Finally, the patterned WGP was diced into individual 2 × 2 mm^2^ chips using a dicing saw (DAD3350, DISCO).

### Fabrication of the bi-cell photodiode (PD)

The bi-cell PD was fabricated with a PIN structure using a conventional 200 mm silicon (Si) process as shown in Fig. S4. Fabrication began with a 200 mm, phosphorus-doped Si wafer (thickness 725 μm, resistivity 10kΩ·cm). An 850 nm-thick thermal SiO_2_ layer was grown (E1200, Centrotherm) and used as an implantation mask. After selective oxide removal (EXELAN-HPT, Lam Research), phosphorus ions were implanted at 80 keV with a dose of 5 × 10^15 ^cm^-2^ to define the cathode region, followed by annealing at 1100 °C for 1 h. Boron implantation was subsequently performed at 80 keV and 5 × 10^14 ^cm^-2^ after etching the oxide layer, and a second annealing at 1100 °C for 1 hour was conducted to form a guard ring structure surrounding the active area, enhancing breakdown voltage. The active region was defined by patterning the SiO₂ film within the guard ring and implanting boron at 10 keV and 5 × 10^14 ^cm^-2^. Dopant activation was achieved via rapid thermal annealing (RTA200H-SP1, NYMTECH) at 1000 °C for 10 s. An anti-reflection (AR) coating of 95 nm SiN_x_ was deposited by plasma-enhanced chemical vapour deposition (PECVD) (P-5000, AMAT) to improve quantum efficiency (QE) in the near-infrared (NIR) region. A metal stack consisting of 100 nm Ti and 1 μm Al was deposited by electron beam evaporation and patterned using a standard lift-off process to form metal contacts. Wafer singulation was achieved using a plasma dicing before grinding (PDBG) process^50,51^. Deep reactive ion etching (DRIE) (Neogen II, Gigalane) to a depth of 170 μm was performed on the front side, with dicing areas defined by photolithography using an i-line contact aligner (EVG640, EVG). After photoresist removal via O_2_ plasma ashing (DAS-2000, PSK) and tape lamination, backside grinding (DGP8760, DISCO) reduced the wafer thickness to 150 μm.

### CSP-iPRD assembly

The bi-cell PD was first die-attached onto a printed circuit board (PCB) and electrically connected via wire bonding using a wedge bonder (Model 5422, Kulicke & Soffa) with 25 μm diameter gold (Au) wire. The 2-in-1 WGP was mounted onto an anodized aluminum spacer, which was precision-machined by CNC milling. The WGP was bonded to the aluminum spacer using a thermally cured epoxy adhesive (Torr Seal, Agilent), followed by curing at 60 °C for 30 min. Subsequently, the spacer with the integrated WGP was carefully aligned and attached onto the PCB using Torr Seal under the same curing conditions to complete the CSP-iPRD assembly.

For system-level evaluation, an additional PCB was fabricated with dimensions compatible with standard 1-inch optical components. The bi-cell PD was assembled on this evaluation PCB following the same die-attach and wire-bonding process using the K&S 5422 bonder with 25 μm Au wire. The anodized aluminum spacer with the integrated 2-in-1 WGP was then aligned and bonded onto the evaluation PCB using Torr Seal adhesive, followed by curing at 60 °C for 30 min, forming the evaluation version of the CSP-iPRD.

### Structural characterization

The structural morphology of the fabricated devices was characterized using scanning electron microscopy (SEM) and transmission electron microscopy (TEM). SEM imaging at 3.0 kV was performed during FIB-based TEM sample preparation (Helios 5 UX, Thermo Fisher Scientific). TEM analysis was conducted at 300 kV (Tecnai G2 F30, FEI).

### Electrical and optical characterization

The current–voltage (I–V) characteristics of the fabricated bi-cell PD were measured under dark conditions using a probe station (PAV200, Formfactor) and a semiconductor parameter analyzer (4200-SCS, Keithley). Voltages ranging from –10 V to +3 V were applied to the anode electrode in 0.5 V increments, and the resulting currents were recorded.

Spectral responsivity (SR) was measured across the visible to near-infrared (NIR) range (400–1000 nm) using a quantum efficiency measurement system (QUANTX-300, Newport). A reverse bias of 5 V was applied during measurement to operate the PD in photocurrent mode. An optical chopper operating at 25 Hz was combined with a lock-in amplifier to suppress external noise and enhance the signal-to-noise ratio.

The polarization properties of the 2-in-1 WGP were evaluated by measuring the transmittance of normally incident TE- and TM-polarized light. The polarization extinction ratio (PER) was calculated by dividing the transmittance of TM-polarized light by that of TE-polarized light. Transmission spectra of the WGP samples were obtained over a wavelength range of 400 nm to 1100 nm, with a step size of 5 nm, using a UV-Vis-NIR spectrophotometer (UH4150, Hitachi). The spectrophotometer is equipped with a deuterium lamp for the UV region and a tungsten-halogen lamp for the visible to near-infrared (Vis-NIR) region, utilizing an integrated double monochromator prism-grating (P-G) optical system. The TE- and TM-polarized incident light was filtered using a half-wave plate, and the transmitted light through the WGP was measured using a 60 mm standard integrating sphere as shown in Fig. S5.

For optical characterization of the 2-in-1 WGP and the CSP-iPRD, an optical setup based on a linearly polarized 795 nm vertical cavity surface-emitting laser (VCSEL; L795VH1, Thorlabs) was used. The VCSEL was mounted in a laser driver mount (LDM56, Thorlabs) and driven by a benchtop laser current controller (LDC205C, Thorlabs). The output beam was collimated using plano-convex and plano-concave lenses with f = 25 mm (LA1951, Thorlabs) and f = -30 mm (LC2679, Thorlabs), respectively, as shown in Fig. S6. Measurements were conducted at optical power levels ranging from 0.12 mW to 0.989 mW, modulated up to 100 kHz.

Spatial polarization transmission was evaluated by directing a linearly polarized 795-nm beam onto the 2-in-1 WGP. The transmitted beam profile was captured using a complementary metal–oxide–semiconductor (CMOS) image sensor (CS165MU1, Thorlabs). Measurements were performed at polarization rotation angles of 0°, 45°, and 90° using a rotational mount (RSP1, Thorlabs). The 2-in-1 WGP exhibited spatial separation of polarization components, selectively transmitting co-polarized light and enabling the measurement of polarization rotation. Beam profiles along the indicated line were analyzed using ImageJ open-source software.

Angular resolution was characterized by directly illuminating the linearly polarized 795-nm laser onto the CSP bi-cell photodiode. The CSP-iPRD device was mounted on a rotational mount (CRM1PT, Thorlabs) and aligned to have uniform optical input. The rotation angle was measured using the Vernier micrometer integrated into the mount. The bi-cell photodiode was interconnected for balanced mode operation, and the current from the balanced detector was measured using a source measurement unit (SMU; B2902A, Keysight).

To evaluate the CMMR of the CSP-iPRD, a sinusoidal modulation signal was generated using a function generator (DG1032Z, Rigol) and applied to the drive current of a 795 nm VCSEL. The CSP-iPRD was aligned to have uniform optical input to both photodiode cells. The CMMR was determined by separately measuring the common-mode and differential-mode output signals using a dynamic signal analyzer (SR785, Stanford Research Systems). Since the analyzer reports magnitude in decibels (dB), the CMMR was calculated as the difference between the measured common-mode and differential-mode output magnitudes in the dB domain:

$${\rm{CMMR}}={V}_{{com}}\left[{dB}\right]-{V}_{{diff}}\left[{dB}\right]$$

### Magnetic field sensitivity measurement integrated with SERF OPM

The SERF magnetometer was implemented on a vibration-isolated optical table using discrete free-space optics. The vapor cell, with dimensions of 1 × 1 × 1 cm^3^, was filled with 700 Torr of nitrogen (N_2_) buffer gas and neutral ^87^Rb atoms. It was enclosed within an oven constructed from hexagonal boron nitride, a non-magnetic material with high thermal conductivity, and polyether ether ketone, known for its high heat resistance and mechanical strength. A high-frequency current at 50 kHz was applied to a heating film in contact with the oven, raising the temperature to 147 °C. This thermal environment prepares the atomic spin state in the SERF regime, allowing for measurement under near-zero magnetic field conditions. The vapor cell was housed inside a cylindrical magnetic shielding box with a shielding factor of 10^6^ to suppress external magnetic field noise. Additionally, a set of three-axis Helmholtz coils was employed to compensate for residual magnetic fields and to control external magnetic fields as needed. In this setup, the minimum detectable magnetic sensitivity was limited to 10 fT/Hz^1/2^ due to Johnson noise originating from the magnetic shielding box itself.

The laser beams incident on the vapor cell were generated by a 795 nm laser tuned to the D1 transition line of ^87^Rb. A circularly polarized pump beam, blue-detuned by 2 GHz from the ^87^Rb D1 line, interacts with the atoms, aligning their spins along the direction of the pump beam propagation. These aligned spins precess in the plane of the laser beams under the influence of an external magnetic field applied along the y-axis (perpendicular to the plane), as shown in Fig. 6a, which results in the polarization rotation of a linearly polarized probe beam. The probe beam, also incident perpendicularly and blue-detuned by 25 GHz from the ^87^Rb D1 line, undergoes Faraday rotation due to the spin precession. After magnetic shielding, a half-wave plate is used to adjust the linear polarization of the probe beam such that it passes through a Wollaston polarizer and splits equally into vertical and horizontal polarization components.

For performance comparison, a conventional detection scheme was also implemented under identical optical conditions. This comparative setup enabled evaluation of the CMMR, angular sensitivity, and packaging advantages of the CSP-iPRD relative to the standard free-space configuration. A flipper mirror was inserted between the Wollaston polarizer and the vapor cell to direct the beam to the fabricated CSP-iPRD (2-in-1 WGP and bi-cell photodiode) in a separate optical path. The CSP-iPRD was mounted directly in the optical path and aligned to receive the two orthogonal polarization components spatially separated by the WGP. The differential output from the CSP-iPRD was routed via an SMA connector to a low-noise current preamplifier (SR570, Stanford Research Systems) for further signal conditioning. The resulting signal was processed using a lock-in amplifier (LIA) and recorded by a data acquisition system (DAQ) for spectral analysis.

## Supplementary information

Supporting Information
