# Supplementary material for: Chip-scale packaged in-line polarization-resolved detector for optically pumped magnetometers
Source: Microsyst Nanoeng. 2026 Mar 31;12:114. doi: 10.1038/s41378-026-01226-z (PMC13035817; doi:10.1038/s41378-026-01226-z)
Supplement: Supplementary file 1 — Supporting Information [file 41378_2026_1226_MOESM1_ESM.docx]

Chip-scale packaged in-line polarization-resolved detector for optically pumped magnetometers

Hui Jae Cho^2,3,†^, Yeeun Na^1,3†^, Sanghyun Park^4,5^, Min-Hwan Lee^4,5^, Hyogi Kim^4,5^, Jihyeong Ju^4,5^, Dooyoung Kim^4,5^, Il-Suk Kang^1^, Jong-Bum You^1^, Gayoung Park^2^, Gapseop Sim^2^, Seung-In Lee^2^, Byung Il Lee^2^, Soo Hyun Kwon^2^, Jong Hyun Song^2^, Eui-Je Jo^2,3^, Tae Hyun Kim^2^, Giung Rang^6^, Jae Hyuk Ahn^3^, Hyoungho Ko^3^, Jongwon Lee^7^, Jong-Kwon Lee^8^, Geol Moon^4,5,*^, and Jongcheol Park^1,*^

# Supporting Information

**Supplementary Results**

**WGP simulation.**To clarify the origin of the wavelength-dependent PER oscillations observed in Fig. 1b, electromagnetic simulations were performed using both ideal rectangular WGP geometries and fabrication-informed geometries incorporating sidewall tapering and top rounding (Fig. S1). These simulations were designed to evaluate the intrinsic optical response of the WGP structure itself and therefore did not include additional experimental factors such as finite substrate thickness, Fabry–Pérot interference, surface roughness, anti-reflection (AR) coatings, or wavelength-dependent material absorption beyond the grating structure.

As shown in Fig. S1, the simulated transmittance spectra reproduce the overall spectral trends observed in the experiment, while the measured absolute transmittance is consistently lower by approximately ~10%. This discrepancy is attributed to additional optical losses introduced by unmodeled experimental factors, including surface scattering, interface reflections, and substrate-related interference effects. The simulations further reveal that fabrication-induced geometric deviations primarily affect the suppression of the cross-polarized transmission, whereas the co-polarized transmission exhibits a similar spectral trend to that of the ideal geometry. Consequently, the apparent enhancement and oscillatory behavior of the PER are dominated by changes in the cross-polarized component rather than improvements in co-polarized transmission.

The large amplitude of the PER oscillations can be understood from the definition PER=Tp/Ts. In the wavelength range where Ts is strongly suppressed, even small absolute variations in Tp or Ts result in amplified fluctuations in the PER. Fig. S1c and Fig. S1d shows that both Tp and Ts exhibit wavelength-dependent variations in the experiment, with the effect being more pronounced for Ts in the low-transmission regime.

In addition, the finite spectral bandwidth of the monochromator-based excitation source leads to spectral averaging effects, which can further modify narrow spectral features arising from interference or absorption. This effect is more pronounced for the cross-polarized transmission and can contribute to enhanced PER fluctuations near transmission minima, particularly in the 800–850 nm wavelength range. These observations are unlikely to arise solely from measurement artifacts; rather reflect the intrinsic sensitivity of ratio-based metrics under low-signal conditions.

Similar non-monotonic PER behavior in the near-infrared region has been reported in previous studies on wire-grid polarizers, indicating that such features are not unique to the present device but represent a general characteristic of WGP structures operating in the low-transmission regime.


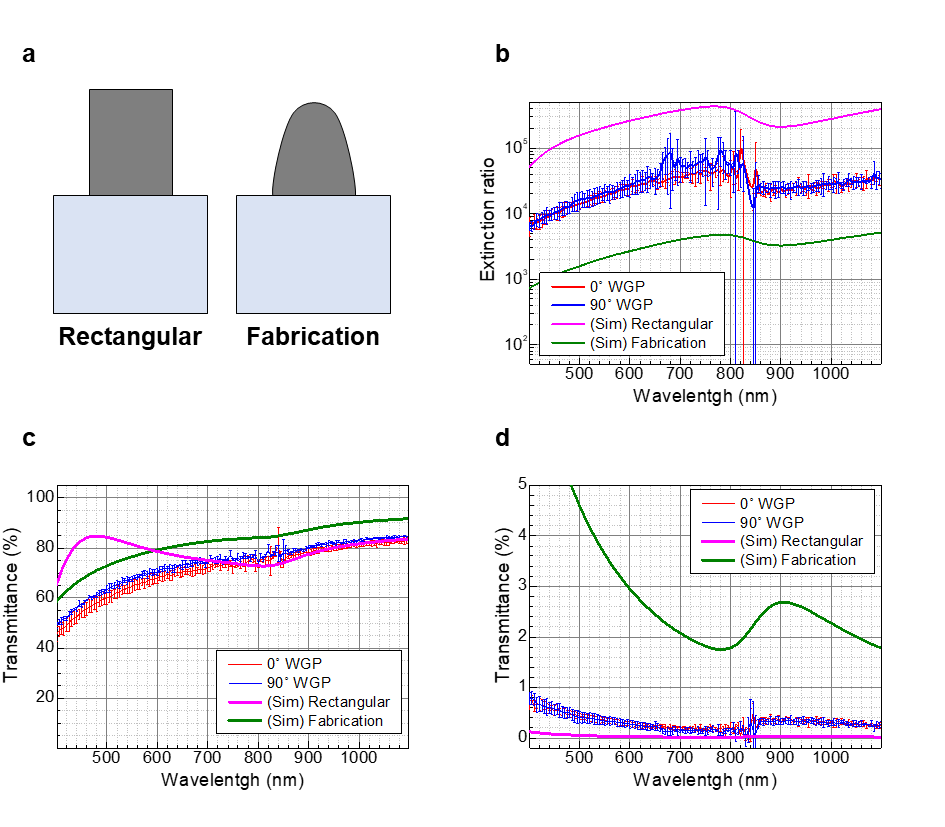


**Fig. S1 Simulation model and wavelength-dependent transmittance and polarization extinction ratio (PER) of the WGP. a** Cross-sectional geometries used in simulations: an ideal rectangular profile and a fabrication-informed profile incorporating sidewall tapering and top rounding. **b** Measured and simulated PER (extinction ratio) as a function of wavelength for 0° and 90° WGP orientations. **c** Measured and simulated co-polarized transmittance versus wavelength. **d** Measured and simulated cross-polarized transmittance versus wavelength. These results illustrate that the apparent PER enhancement primarily originates from the cross-polarized transmission approaching the measurement noise floor.

**Electrical measurement of bi-cell photodiode.** To evaluate the electrical matching between the two photodiode units forming the bi-cell detector, the current–voltage (I–V) characteristics of Cell A and Cell B were measured individually. As shown in Fig. S2a, the two cells exhibit nearly identical I–V behavior over the full bias range, with closely matched dark current levels and turn-on characteristics. In particular, near zero bias, the dark currents of the two cells are nearly indistinguishable, confirming excellent electrical matching. This high degree of matching is essential for achieving effective common-mode rejection and low-noise differential operation in the CSP-iPRD.

To verify that CSP integration does not deteriorate common-mode rejection performance, both the bare bi-cell photodiode and the CSP-iPRD were tested using the same optical and electrical setup. Supplementary Fig. S2b shows that the differential-mode signals remain consistently suppressed across the frequency range, with minimal variation between the bare and CSP configurations.


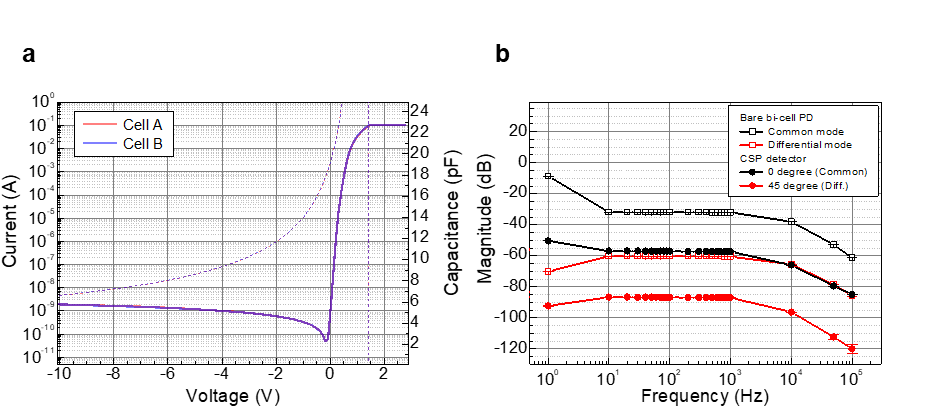


**Fig. S2 Electrical characterization and matching of the bi-cell photodiode.** **a** Current–voltage (I–V) and capacitance–voltage (C–V) characteristics of the two photodiode units (Cell A and Cell B) forming the bi-cell detector, measured individually under identical conditions. The I–V curves (left axis) exhibit nearly overlapping behavior over the entire bias range, indicating closely matched dark current characteristics. The C–V curves (right axis) show similar capacitance values and voltage dependence for both cells. In particular, near zero bias, both the dark current and junction capacitance are nearly indistinguishable between the two photodiode units, confirming excellent electrical matching. Such matching is critical for achieving high common-mode rejection and low-noise differential operation in the CSP-iPRD. **b** Frequency-dependent output signals of the bare bi-cell photodiode and the CSP-iPRD measured using a dynamic signal analyzer. Both common-mode (black) and differential-mode (red) responses were measured over the 1 Hz to 100 kHz modulation frequency range for the bare bi-cell photodiode (open rectangles) and the CSP-iPRD (filled circles). The CSP-iPRD exhibits comparable suppression of differential-mode signals relative to the bare photodiode, confirming that chip-scale packaging does not degrade common-mode rejection performance. The signal roll-off observed above ~80 kHz is attributed to the bandwidth limitation of the laser diode current driver.

To evaluate the effect of optical power on angular sensitivity, the differential photocurrent of the CSP-iPRD was measured at several incident power levels ranging from 41.4 μW to 84.9 μW. The angular resolution was extracted by fitting the linear portion of the response curve as a function of polarization rotation angle. Supplementary Table S1 summarizes the extracted angular resolution, normalized sensitivity per optical power, and R^2^ values for each measurement. The high R² values (0.994–0.999) confirm the excellent linearity of the detector response, even under varying illumination conditions.

Using the measured angular responsivity (dI/dθ) and the input current noise of the front-end TIA (i_n_=5 pA/Hz^1/2^), the corresponding noise-equivalent angular resolution is estimated as

$$\delta\theta=\frac{i_{n}}{{dI}/{d\theta}}$$

Here, the TIA input current noise is taken from the manufacturer datasheet, and the angular resolution is expressed as a noise-equivalent value per Hz^1/2^.

**Table S1** Angular resolution of the CSP-iPRD under varying optical power

| Input power (μW) | Angular responsivity (nA/deg) | Angular resolution  (degree/Hz^1/2^) | Normalized sensitivity (nA/deg/μW) | R^2^ |
| --- | --- | --- | --- | --- |
| 41.4 | 27.88 | 1.8 × 10^-4^ | 0.673 | 0.995 |
| 65.6 | 37.37 | 1.3 × 10^-4^ | 0.569 | 0.994 |
| 84.9 | 59.31 | 8.4 × 10^-5^ | 0.698 | 0.999 |

To verify that the measured angular response originates from the intended polarization-selective detection rather than beam-profile artifacts, we additionally characterized devices with the 2-in-1 WGP mounted in the flipped orientation (i.e., rotated by 180° relative to the detector) as shown in Fig. S3. As expected from the bi-cell differential readout geometry, flipping the WGP reverses the sign of the angular responsivity while preserving the magnitude and linearity of the response. The measured responses exhibit consistent slopes with opposite signs across multiple assemblies, the absolute angular responsivity was ∣dI/dθ∣=74.9±7.2 nA/deg (mean ± s.d.), confirming good reproducibility and indicating that the angular sensitivity is not dominated by interference-related artifacts

**Fig. S3 Angular response reproducibility under flipped WGP orientation.** The differential photocurrent exhibits linear dependence on polarization rotation angle. Flipping the 2-in-1 WGP reverses the slope sign while maintaining a comparable magnitude. The mean absolute angular responsivity across multiple assemblies is ∣dI/dθ∣=74.9±7.2 nA/deg.

**Supplementary Method**

**Fabrication of bi-cell photodiode.**A 200 mm high-resistivity silicon wafer was processed using standard CMOS-compatible steps including thermal oxidation, ion implantation, and metallization. To realize the thin bi-cell structure, plasma dicing before grinding (PDBG) was used, enabling precise singulation and thinning down to 150 μm thickness.

**
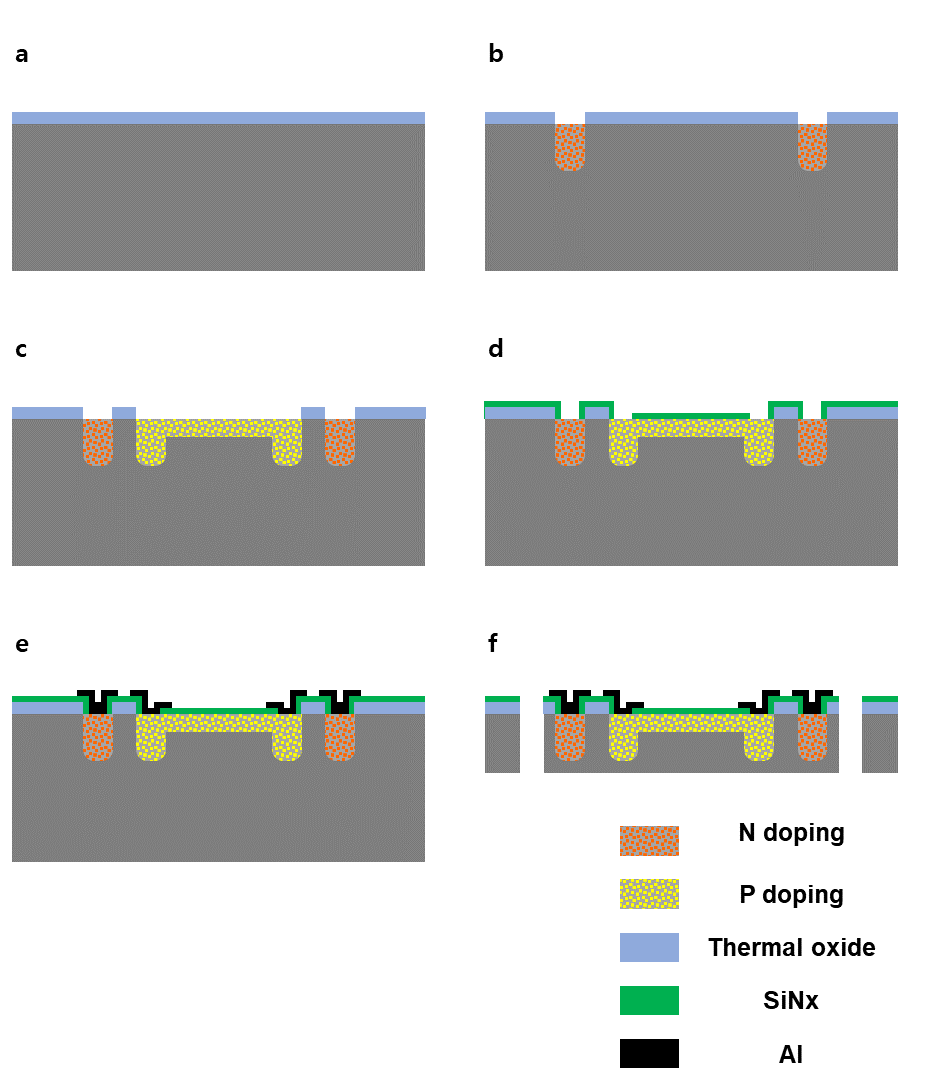
**

**Fig. S4** **Fabrication process flow of the bi-cell photodiode. a** Thermal oxidation of a high-resistance n-type Si wafer to form an 8500 Å-thick SiO₂ layer. **b** Phosphorus ion implantation at 80 keV with a dose of 5 × 10¹⁵ cm⁻² to form the cathode regions. **c** Boron ion implantation for the guard ring (80 keV, 5 × 10¹⁴ cm⁻²) and the active area (10 keV, 5 × 10¹⁴ cm⁻²). **d** Deposition of a 95-nm-thick PECVD SiNₓ anti-reflection layer and via patterning. **e** Metallization using a lift-off process with a 100-nm-thick Ti adhesion layer and 1-μm-thick Al. **f** Plasma dicing before grinding (PDBG) process to obtain octagonal chips with 150 μm thickness.

**Optical setup.**The measurement configuration for spectral transmittance and PER of the WGP is shown in Supplementary Fig. S5. A broadband light source is linearly polarized and filtered using a half-wave plate, and the transmitted intensity is measured using a spectrophotometer with an integrating sphere.

**
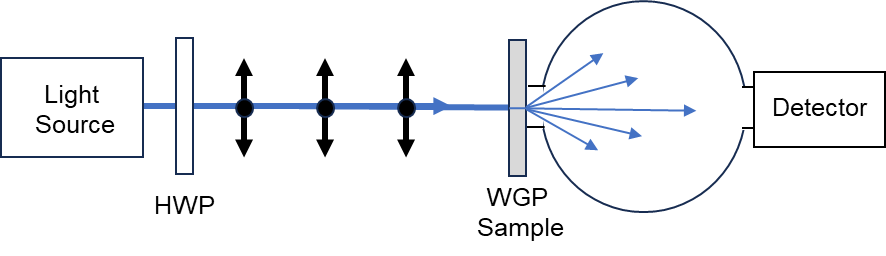
**

**Fig. S5 Optical setup for characterization of the WGP.** A UV-Vis-NIR spectrophotometer equipped with dual light sources and polarization optics is used to measure the transmission spectra. A linear polarizer and a half-wave plate are used to generate TE and TM polarizations. The transmitted light is collected by a 60 mm integrating sphere and directed to the detector.

During beam collimation using a convex lens (f = +25.4 mm) and a concave lens (f = –30 mm), Newton ring interference fringes were observed near the beam axis. The lenses used in the setup were uncoated, and the observed fringes are attributed to interference between partially reflected beams at parallel optical surfaces as shown in Fig S6b and S6c. Given the high temporal and spatial coherence of the single-mode VCSEL source, such interference is expected. The appearance of these fringes does not indicate imperfect collimation and had no measurable impact on the experimental results. The diameter of the collimated beam was estimated to be approximately 4.25 mm, as measured using a CMOS image sensor (CIS).

Accordingly, the Newton’s rings observed in Figs. 2c and 2d and the associated intensity fluctuations originate from the combination of a highly coherent VCSEL source and uncoated collimation optics. These interference effects are therefore unrelated to the device performance and do not affect the quantitative polarization extinction ratio or angular sensitivity measurements reported in the main text.

**
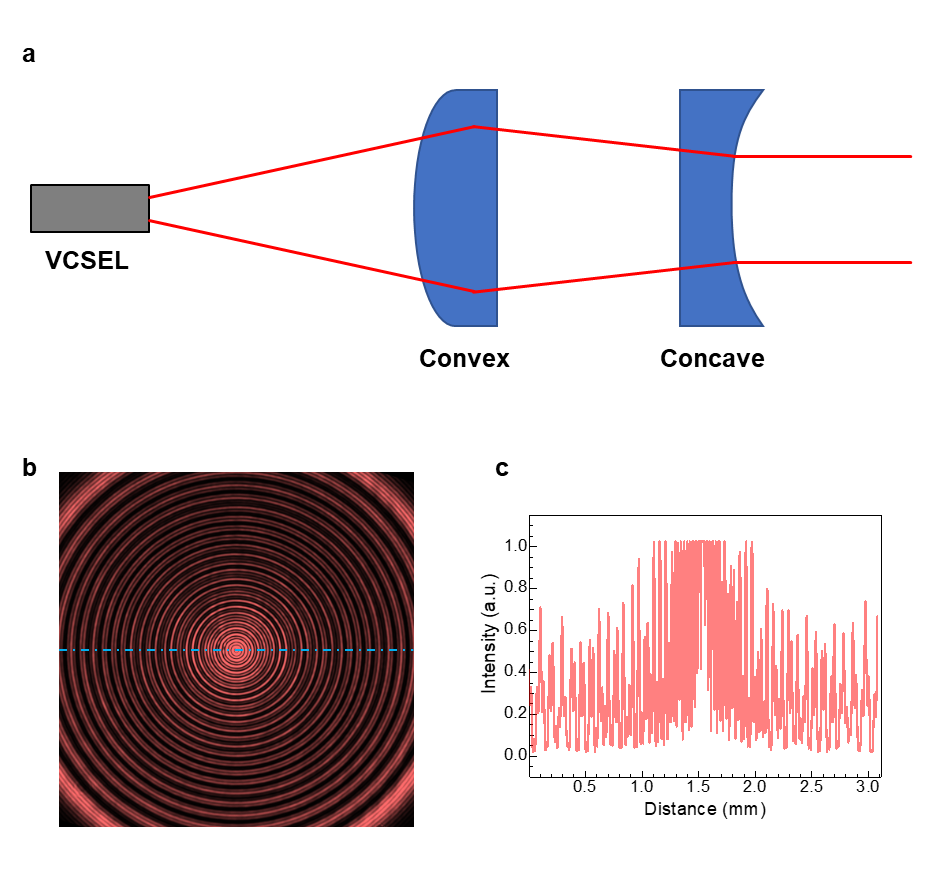
**

**Fig. S6 Optical setup and characterization of the collimated VCSEL beam.** **a** Schematic of the optical setup used to collimate the VCSEL beam, consisting of a convex lens (f = +25.4 mm) and a concave lens (f = –30 mm) separated by approximately 4.6 mm. **b** Newton ring interference pattern captured at the output. The rings result from the high coherence of the single-mode VCSEL source and the use of uncoated optical components. **c** Intensity profile of the collimated beam extracted along the horizontal axis (dashed blue line in **b**), showing a symmetric distribution with a full width of approximately 4.25 mm. The interference does not affect beam quality or experimental performance.
